# The SseC translocon component in *Salmonella enterica* serovar Typhimurium is chaperoned by SscA

**DOI:** 10.1186/1471-2180-13-221

**Published:** 2013-10-04

**Authors:** Colin A Cooper, David T Mulder, Sarah E Allison, Ana Victoria C Pilar, Brian K Coombes

**Affiliations:** 1 Michael G. DeGroote Institute for Infectious Disease Research, Hamilton, Ontario L8N 3Z5, Canada; 2 Department of Biochemistry and Biomedical Sciences, McMaster University, Health Sciences Centre Room 4H17, Hamilton, Ontario L8S 4K1, Canada; 3 Department of Molecular and Cellular Biology, University of Guelph, 488 Gordon Street, Science Complex, Room 4202, Guelph, Ontario N1G 2W1, Canada

**Keywords:** Salmonella, Pathogenesis, Chaperone, Translocon, T3SS

## Abstract

**Background:**

*Salmonella enterica* is a causative agent of foodborne gastroenteritis and the systemic disease known as typhoid fever. This bacterium uses two type three secretion systems (T3SSs) to translocate protein effectors into host cells to manipulate cellular function. *Salmonella* pathogenicity island (SPI)-2 encodes a T3SS required for intracellular survival of the pathogen. Genes in SPI-2 include apparatus components, secreted effectors and chaperones that bind to secreted cargo to coordinate their release from the bacterial cell. Although the effector repertoire secreted by the SPI-2 T3SS is large, only three virulence-associated chaperones have been characterized.

**Results:**

Here we report that SscA is the chaperone for the SseC translocon component. We show that SscA and SseC interact in bacterial cells and that deletion of *sscA* results in a loss of SseC secretion, which compromises intracellular replication and leads to a loss of competitive fitness in mice.

**Conclusions:**

This work completes the characterization of the chaperone complement within SPI-2 and identifies SscA as the chaperone for the SseC translocon.

## Background

Bacterial pathogens exploit host niches using strategies that block or modify host defense pathways. One such strategy employed by the Gram-negative bacterium *Salmonella enterica*, is the translocation of effector proteins into the host cell through a type three secretion system (T3SS). *S. enterica* serovar Typhimurium (*S.* Typhimurium) has two T3SSs encoded within *Salmonella* pathogenicity island-1 (SPI-1) and SPI-2 that facilitate invasion and intracellular survival within host cells [1-3]. The assembly of the T3SS is complex, involving the formation of membrane channels in the bacterial inner and outer membrane, and a terminal translocon that forms a pore in host membranes.

Both SPI-1 and SPI-2 encode a distinct group of chaperones that bind to their cognate cargo proteins to coordinate T3SS assembly and secretion of effectors. Virulence chaperones belong to one of three defined classes [4]: class I chaperones bind to single (IA) or multiple (IB) effectors, class II chaperones interact with translocon components, and class III chaperones partner with apparatus components. Among each of the different classes, chaperones share structural similarity yet their amino acid sequence can be poorly conserved. As such, many chaperones have been first identified based on low sequence identity with previously characterized proteins, and by shared physical properties such as isoelectric point (pI). Class I chaperones tend to be small proteins (~9-15 kDa) with acidic pI, and function as dimers adopting a horseshoe-like shape [5-7]. Class II chaperones also form dimers but do not have an acidic pI, which reflects a different interaction surface required for substrate binding [8,9].

In addition to directing secretion events, chaperone-cargo pairs can function as regulatory proteins for T3SS gene expression [10]. The FlgN chaperone interacts with FlgK-FlgL to form a repressive complex that inhibits expression of late flagellar genes [11]. The *Yersinia* virulence plasmid encodes the chaperone SycD (also known as LcrH) that aids in the secretion of the translocon components YopB and YopD, the latter of which is responsible for establishing a negative feedback loop to prevent effector gene expression at early stages of infection [12]. As SycD is required for YopD stability in the cytosol, both chaperone and cargo are necessary for proper coordination of Yop expression.

In *S. enterica*, over twenty effectors secreted by the SPI-2 T3SS have been identified yet the full complement of virulence chaperones involved in their secretion remains to be identified or functionally analyzed. To date, three virulence chaperones have been characterized; we showed that SrcA chaperones the effectors SseL and PipB2 and binds to the T3SS ATPase SsaN [5]. The SscB chaperone directs the secretion of SseF [13], and the class II chaperone, SseA, is responsible for the secretion of the putative translocon platform protein SseB and one of the two translocon proteins, SseD, but not SseC [14-16]. Comparative sequence analysis of SPI-2 identified a putative chaperone gene called *sscA*[17] but its function had yet to be demonstrated. In light of these findings, we set out to identify and characterize the chaperone involved in secretion of the SseC translocon protein, with an *a priori* focus on the *sscA* gene in SPI-2. In this study we demonstrate that SscA interacts with SseC and is required for its secretion but is dispensable for secretion of the other translocon components SseD and SseB. Both SscA and SseC were required for fitness in infected mice and *in vitro* macrophage infection assays.

## Results

### Identification of SscA as a chaperone for SseC

**Figure 1:**
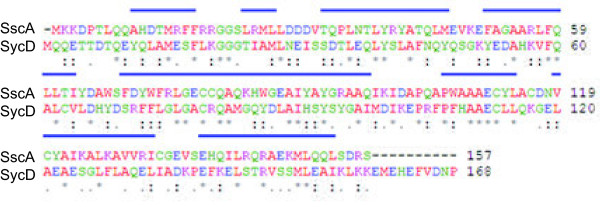
**Amino acid sequence alignment of SscA and the *Yersinia *chaperone SycD.** Conserved alpha helical regions are denoted with blue bars. Alignment was performed with Clustal W software (http://www.ebi.ac.uk), alpha helix content was inferred from the published SycD crystal structure (PDB 2VGY) and from predictions made using SSpro8 [21].

SscA was previously predicted to be a chaperone based on comparisons to other T3SS-associated chaperones and therefore we prioritized it for analysis [17]. SscA is an ~18 kDa protein that has 46% sequence identity to SycD, a translocon chaperone in *Yersinia*. Using the SycD crystal structure as a model (PDB-2VGY), the secondary structure prediction for SscA [18] showed a solely α-helical protein consisting of eight α-helices and a large tetratricopeptide repeat (TPR) domain from amino acids 36 to 137 (Figure 1). This helical structure is similar to that found in SycD [8] while the TPR domain has been shown in mutational studies and structural work to be involved in cargo binding for class II chaperones [19,20]. Based on this structural comparison, we aimed to further characterize SscA as a potential class II chaperone in the SPI-2 T3SS.

### SscA interacts with the translocon protein SseC

**Figure 2:**
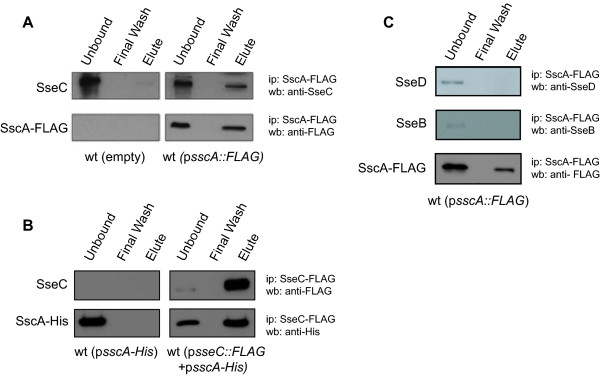
**SscA interacts with the translocon protein SseC. (A)** Wild type *Salmonella* (left panels) and a strain carrying a plasmid expressing SscA-FLAG (right panels) were grown in LPM minimal medium, lysed and subjected to immunoprecipitation with anti-FLAG antibody. Immunoprecipated proteins were probed by Western blot with anti-SseC antiserum and anti-FLAG antibody. **(B)** A reciprocal immunoprecipitation to that shown in part A was performed with a strain expressing SscA-His_6_ and a strain expressing both SscA-His_6_ and SseC-FLAG as indicated. SseC-FLAG was immunoprecipitated and proteins were blotted using anti-His and anti-FLAG antibodies. **(C)** SscA-FLAG does not immunoprecipitate the SseB or SseD translocon proteins. The specificity of the SscA-SseC interaction was tested by probing SscA-FLAG immunoprecipitates with antibodies raised against SseD and SseB, neither of which was detectable in the final eluted protein fraction. Each immunoprecipitation experiment was repeated three times with similar results.

Chaperones exert their biological function in T3SS export through a physical interaction with cargo proteins. Because the class II chaperone SseA binds the translocon components SseB and SseD but not SseC, this suggested that another chaperone interacts with SseC. The structural similarity of SscA to SycD made this protein a logical candidate. To test this hypothesis we immunoprecipitated SscA-FLAG from bacterial cells and analyzed the co-precipitated proteins by Western blot with anti-SseC antiserum. SseC was pulled down only in the *Salmonella* strain expressing SscA-FLAG and not from control lysates generated from untagged wild type cells (Figure 2A). To verify this interaction, we performed a reciprocal co-IP by pulling down SseC-FLAG and showing co-precipitation of SscA-His_6_ in the eluted protein fraction (Figure 2B). To examine the specificity of the SscA-SseC interaction, we tested whether SscA-FLAG could immunoprecipitate other members of the translocon apparatus, including SseB and SseD, which it did not (Figure 2C). These data indicated that SscA interacted with SseC, but not the other translocon proteins.

### SscA is necessary for secretion of SseC

**Figure 3:**
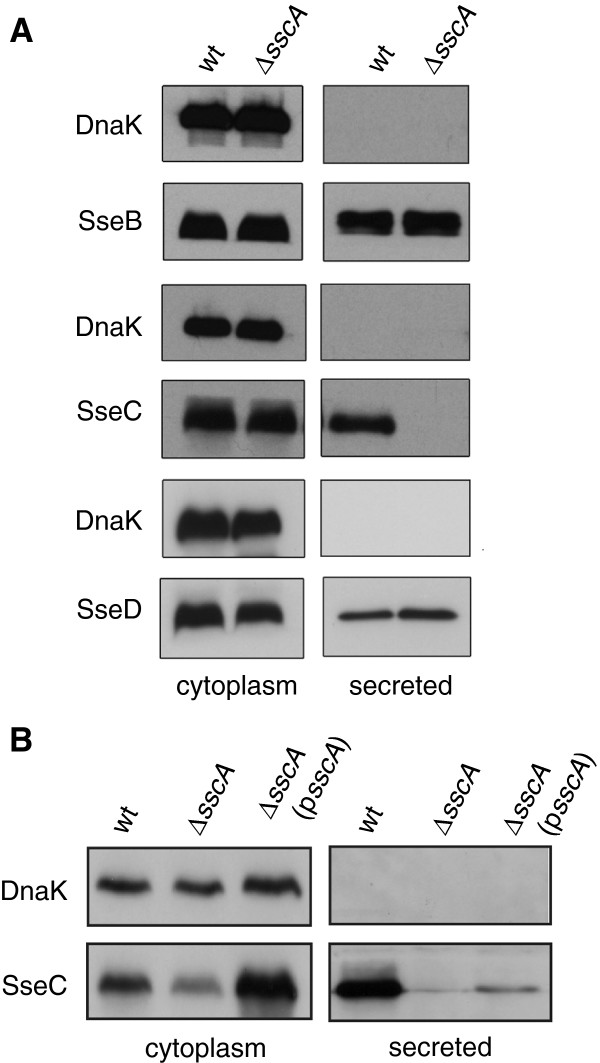
**SscA is required for the secretion of SseC. (A)** Proteins isolated from the cytoplasm and those secreted into the culture medium by wt and an ∆*sscA* mutant were probed by Western blot for the translocon components SseB, SseC and SseD. All proteins were detected in the cytoplasmic fraction from both strains. Wild type cells secreted each of the translocator apparatus proteins, however, SseC was undetectable in the secreted fraction from ∆*sscA* with no affect on SseB or SseD. Anti-DnaK antibody was used as a control to verify the absence of cytoplasmic protein in the secreted protein fractions. **(B)** Complementation of ∆*sscA* modestly restores SseC secretion. Whole cell lysates and secreted protein fractions from wild type, ∆*sscA,* and ∆*sscA* transformed with a plasmid encoding *sscA* were probed for SseC by Western blot. SseC was detected in the secreted fraction from complemented ∆*sscA,* albeit to lower levels than that seen from wild type cells. Secretion experiments were performed three times with similar results.

To determine if the interaction between SscA and SseC was necessary for SseC secretion, we performed an *in vitro* secretion assay using wild type and Δ*sscA* under conditions that activate expression and activity of the SPI-2 T3SS. The secreted protein fraction from the culture supernatant of both wild type *S*. Typhimurium and Δ*sscA* was immunoblotted for the translocon proteins SseB, SseC, and SseD using specific antisera. The *sscA* mutant failed to secrete SseC as this protein was absent from the secreted protein fraction despite abundant levels in the bacterial cytoplasmic fraction (Figure 3A). SseC was detected in both the secreted protein and cytoplasmic fractions from wild type *Salmonella* and deletion of *sscA* had no demonstrable effect on the secretion of SseB or SseD (Figure 3A). To verify this phenotype, we complemented the Δ*sscA* mutant by transforming it with a plasmid to restore *sscA* expression. This lead to detectable levels of SseC in the secreted protein fraction, albeit to lower levels than wild type (Figure 3B), likely due to the increased copy number of SscA relative to its SseC cargo.

### SseC and SscA are required for fitness during infection

**Figure 4:**
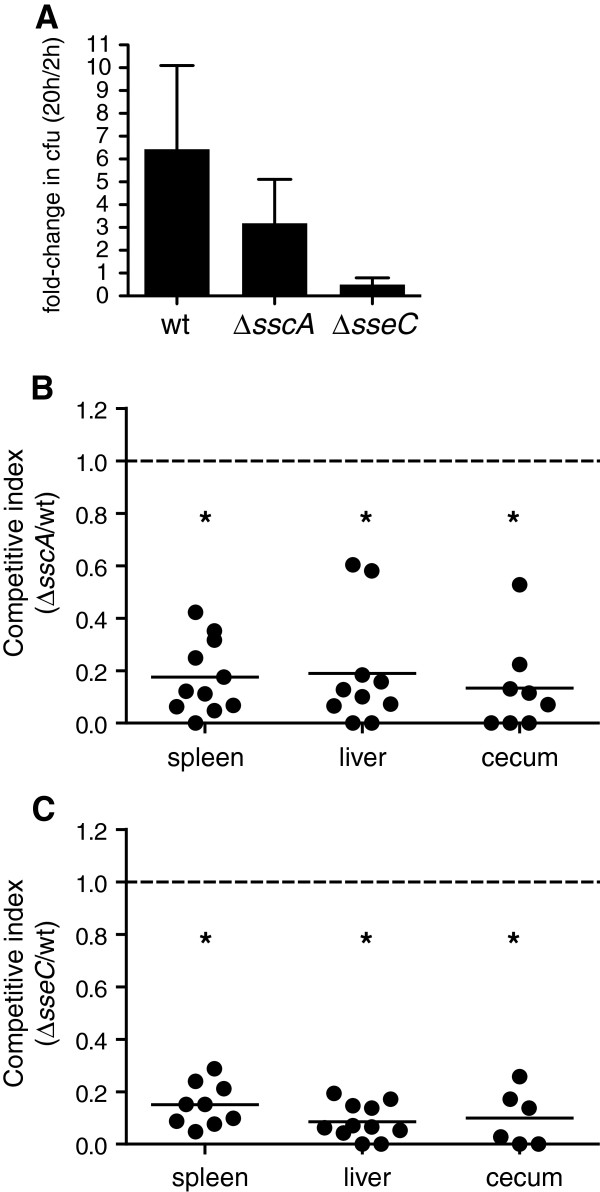
**SscA and SseC are required for fitness during infection. (A)** RAW 264.7 cells were infected with wild type, ∆*sscA* or ∆*sseC* mutant *S.* Typhimurium and the change in intracellular bacteria numbers between 2 h and 20 h post-infection was determined in gentamicin protection experiments. Data are expressed as the mean with standard error of three separate experiments. The competitive index of the ∆*sscA* mutant **(B)** and ∆*sseC* mutant **(C)** was determined in the spleen, liver and cecum 72 h after infection of C57BL/6 mice. Each data point represents an individual animal and data is from two separate experiments. *, p*<*0.05.

Given that SscA was required for secretion of the SseC translocon component, we measured the impact on bacterial fitness following the deletion of *sseC* and *sscA.* Deletion of either *sscA* or *sseC* reduced the ability of bacteria to survive in RAW264.7 macrophages compared to wild type (Figure 4A). The number of intracellular bacteria between 2 h and 20 h after infection was decreased to 10% of wild type in the *sseC* mutant, and to 50% of wild type in the *sscA* mutant. To determine whether similar phenotypes could be observed in animal infections, mice were orally gavaged with a mixed inoculum containing equal proportions of wild type and mutant bacteria and the competitive fitness was determined 3 days after infection in the spleen, liver and cecum. The competitive indices for both *sseC* and *sscA* mutant strains was below 0.20 and were statistically significant (Figure 4B and 4C). The CI for the *sscA* mutant was 0.18 (95% CI 0.08-0.27; spleen), 0.19 (95% CI 0.31-0.35; liver), and 0.13 (95% CI -0.01-0.20; cecum). Values for the *sseC* mutant were 0.15 (95% CI 0.09-0.21; spleen), 0.09 (95% CI 0.04-0.13; liver), and 0.10 (95% CI -0.01-0.20; cecum). These results indicated that both SseC and SscA are critical for infection of macrophages and for competitive fitness in animals.

## Discussion

Protein-chaperone interactions are essential for T3SS function because they coordinate the delivery and secretion of substrate cargo. Class II virulence chaperones are particularly important since they direct translocon secretion as a prerequisite step for the proper delivery of all subsequent effectors into the host cell. Given the modest sequence similarity between the *Yersinia* class II virulence chaperone SycD and SscA, we analyzed SscA as the potential chaperone for the SseC translocon in the *Salmonella* SPI-2 T3SS. The structure of SycD shows a crescent shape molecule with the concave face possessing protein interaction sites that are common between SycD and SscA (i.e. Y40, Y52, Y93) [8]. The *Shigella* class II chaperone IpgC possesses a similar structure with the concave face binding an amino acid region of its cognate cargo IpaD [22], suggesting that a common cargo-binding region may exist among class II virulence chaperones.

Using protein-protein interaction studies and secretion assays we demonstrated that SscA is the class II virulence chaperone for SseC and showed that this interaction is important for *Salmonella* pathogenicity as deletion of either *sscA* or *sseC* lead to similar attenuated phenotypes in mouse infections. As documented previously, effectors can be secreted to the cell surface of the bacteria in the absence of a functional translocon, however delivery of effector proteins into host cells requires an assembled translocon apparatus [23,24]. Interestingly, the *sseC* mutant had a more pronounced negative effect on replication in RAW264.7 cells suggesting an additional role for SseC that does not depend on its secretion, or that a very small number of bacteria assemble a functional translocon in the absence of the SscA chaperone, allowing for some measure of phenotype recovery *in vitro*. This latter possibility was suggested for *Yersinia* LcrH point mutants that had reduced secretion of translocon proteins but retained some ability to intoxicate host cells from a minimal number of T3SS [25]. In our system, we find this possibility unlikely because we found no evidence for SseC secretion in the absence of SscA chaperone even for highly concentrated samples, and the attenuation level of the *sscA* and *sseC* mutants was similar in animal infections.

## Methods

### Ethics statement

All experiments with animals were conducted according to guidelines set by the Canadian Council on Animal Care. The local animal ethics committee, the Animal Review Ethics Board at McMaster University, approved all protocols developed for this work.

### Bacterial strains, cloning, and growth conditions

*Salmonella enterica* serovar Typhimurium strain SL1344 (*S.* Typhimurium) was used as the wild type parent strain for all mutants generated in this study. Genomic mutations were made using the lambda red mutagenesis method [26]. Electrocompetent cells containing the helper plasmid pKD46 were transformed with PCR-amplified linear DNA cassettes encoding the kanamycin resistance gene from the template plasmid pKD4 and flanked with 50 bp ends homologous for the targeted gene. Successful recombinants were recovered by antibiotic selection and screened by PCR for correct gene disruption. To generate FLAG-epitope fusion proteins for immunoprecipitation experiments, the full-length *sscA* gene was cloned into the plasmid pFLAG-CTC (Sigma) as a HindIII/SalI fragment and full-length *sseC* was cloned as an NdeI/SalI fragment using the primers listed in Table 1. Expression was induced from P_*tac*_ using IPTG. To generate an *sscA* fusion to the hexa-histidine (His_6_) epitope, full-length *sscA* was amplified from chromosomal SL1344 DNA using primers that incorporated the His_6_ epitope (Table 1). This fragment was cloned first into pBAD24 [27] as an NheI/HindIII fragment and then subcloned along with P_BAD_ into pACYC (New England Biolabs) as a BamHI/HindIII fragment to generate an arabinose-inducible *sscA-His*_*6*_ fusion. Strains, plasmids and primer sequences used in this work are provided in Table 1. The minimal medium LPM at pH 5.8 (5 mM KCl, 7.5 mM (NH_4_)_2_SO_4_, 0.5 mM K_2_SO_4_, 80 mM MES, 38 mM glycerol, 0.1% casamino acids, 8 μM MgCl_2_, 337 μM PO_4_^3-^), was used to induce the expression of SsrB-regulated genes as described previously [28]. Bacterial cultures were grown at 37°C with shaking at 225 rpm unless otherwise indicated.

**Table 1:** Strains, plasmids and oligonucleotides used in this study

**Strains, plasmids and primers**	**Description**	**Reference or source**
**Strains**		
SL1344	*Salmonella enterica* serovar Typhimurium wild type	Our collection
∆*sscA*	SL1344, deletion of *sscA*	This study
∆*sseC*	SL1344, deletion of *sseC*	This study
SL1344 RES	SL1344, *ushA*::*Cm;* Cm^R^ (10 μg/ml)	[29]
**Plasmids**		
pKD4	oriRγ, Kan^R^ cassette flanked by FRT sites	[26]
pKD46	RepA1019(Ts), λ, γ, β and *exo* expressed from P*araBAD*, Amp^R^	[26]
pFLAG-CTC	Cytoplasmic expression of C-terminal FLAG fusion protein under control of the P*tac*, Amp^R^	Sigma
p*sscA-FLAG*	pFLAG-CTC, expresses in-frame fusion of SscA-FLAG	This study
p*sseC-FLAG*	pFLAG-CTC, expresses in-frame fusion of SseC-FLAG	This study
pACYC184	General cloning, Tet^R^, Cm^R^	Our collection
p*sscA-6His*	pACYC; expresses in-frame fusion of SscA-6His	This study
pBAD24	Arabinose-inducible gene expression, pBR322*ori,* P_BAD_, *rrnBT*_1,2_, *araC*, Amp^R^	[27]
p*sscA*	*sseA-sscA* with endogenous *sseA* promoter in pWSK29, pSC101*ori*, Amp^R^	This study
**Primers**		
*sseC lambda-F*	cgaattcacagtaatagcgacagcgccgcaggagtaaccgccttaacacagtgtaggctggagctgcttcg	
*sseC lambda-R*	gcgatagccagctattctcgcctgaacgctactatagtgatcaatggtatcatatgaatatcctcctta	
*sscA lambda-F*	gacccgaccctacaacaggcacatgacacgatgcggtttttccggcgtgggtgtaggctggagctgcttcg	
*sscA lambda-R*	gtcagaaagttgctgtaacatcttttctgcacgctgtcggagaatttgatcatatgaatatcctcctta	
*sscA-FLAG-F*	gcttaaagcttatgaaaaaagacccgaccct	
*sscA-FLAG-R*	tatctgtcgacgctcctgtcagaaagttgct	
*sseC-FLAG-F*	ggtcacatatgatgaatcgaattcacagtaa	
*sseC-FLAG-R*	ggtcagtcgacagcgcgatagccagctattc	
*sscA-HIS-F*	gtcaggctagcaggaggatgcatcaccatcaccatcacatgaaaaaagacccgaccc	
*sscA-HIS-R*	gtcagaagcttttagctcctgtcagaaagttg	
*sscA-F*	acgcgtcgacacaggatccgcagcaatatc	
*sscA-R*	gctctagacccctaaatatgcaggctca	

### Immunoprecipitation experiments

Co-immunoprecipitations (co-IP) were performed with *S*. Typhimurium expressing SscA-FLAG or SseC-FLAG from the IPTG-inducible pFLAG-CTC plasmid. A strain carrying empty plasmid was used as a control. Strains were grown overnight in Luria-Bertani broth (LB) and sub-cultured 1:50 into 50 ml of LPM medium and grown to an optical density (OD_600_) of 0.6 at 37°C. Cultures were then centrifuged at 3000 × *g* for 10 min, and re-suspended in phosphate buffered saline (PBS) containing mini-EDTA protease inhibitor cocktail (PBS-PI; Roche). Cells were lysed by 6 pulses of sonication for 30 sec each, with 60 sec intervals between sonication (Misonix Sonicator 3000, Misonix). Lysates were centrifuged at 3000 × *g* for 15 min at 4°C and the supernatant removed to obtain the cytosolic protein fraction. M2-agarose beads conjugated with anti-FLAG antibodies (F-gel; Sigma) was equilibrated with PBS-PI containing 10 μg/ml bovine serum albumin (BSA) for 60 min at 4°C with rocking and washed with PBS-PI three times. The beads were mixed with the cytosolic protein fractions and incubated for 16 h at 4°C with end-on-end mixing. Unbound proteins were removed by centrifuging the F-gel at 1000 × *g* for 5 min and removing the supernatant. The F-gel was washed ten times with PBS-PI containing 0.1% Triton-X100 before eluting bound proteins into sodium dodecyl sulfate (SDS)-sample buffer (1M Tris pH 8.0, 20% SDS, 0.5 M EDTA pH 8, 10% glycerol, 200 mM dithiothreitol). Bound proteins were resolved by SDS-PAGE and transferred to polyvinylidene difluoride membranes (Bio-Rad). Western blots were probed with antibodies to SseC (a gift from Dr. Michael Hensel), the FLAG epitope (Sigma), or the His_6_ tag (Qiagen). For reciprocal co-immunoprecipitations, a strain containing a plasmid encoding *sscA-HIS*_*6*_ and a second compatible plasmid encoding *sseC-FLAG* was used. SscA-His_6_ was induced with arabinose and SseC-FLAG was induced with IPTG as above. In this experiment, the anti-FLAG gel was used for immunoprecipitations and anti-His antibody used in immunoblotting as described above.

### Protein secretion assasy

Wild type *S.* Typhimurium and Δ*sscA* strains were grown overnight in LB and sub-cultured 1:50 into LPM and grown to OD_600_ of 0.6. Cultures were then centrifuged for 2 min at 10,000 × *g* and the supernatant was filtered through a 0.2 μm filter (Pall Scientific) and precipitated with 10% trichloroacetic acid (TCA). Precipitated secreted proteins were centrifuged at 16,000 × *g* at 4°C for 30 min and the pellets were washed with acetone and dissolved in SDS-sample buffer. The whole cell lysate fraction was made by dissolving the bacterial pellet in SDS-sample buffer. To ensure equal protein loading in SDS-PAGE, the volume of SDS sample buffer was adjusted according to the optical density of the original culture. Protein samples were analyzed by Western blot using antibodies to DnaK (Convance), SseC (a gift from Dr. Michael Hensel), SseB, SseD, and SseG (a gift from Dr. John Brumell).

### Macrophage replication assays

RAW264.7 cells were grown in Dulbecco’s Modified Eagle Medium (DMEM; Gibco) supplemented with 10% fetal bovine serum (FBS; Invitrogen) at 37°C with 5% CO_2_. Cells were seeded 16 h prior to infection into 24-well plates at a density of 2 × 10^5^ cells per well. Overnight cultures of bacteria were washed with PBS, diluted in DMEM/10% FBS, and used to infect macrophages at a multiplicity of infection (MOI) of 50 for 30 min at 37°C, 5% CO_2._ Infected cells were washed three times with PBS and the media was replaced with DMEM/10% FBS/100 μg/mL gentamicin for 1.5 h to kill extracellular bacteria. Cells were then washed twice with PBS and incubated for 20 h in DMEM/10% FBS with10 μg/mL gentamicin. At 2 h and 20 h after infection, the cells were washed twice with PBS then lysed with 1% Triton X-100, 0.1% SDS in PBS to release intracellular bacteria. Colony forming units (cfu) were determined by plating serially-diluted lysates onto LB agar plates containing appropriate antibiotics. Experiments were performed twice independently using 3 technical replicates per assay.

### Mouse infections

Competitive infections were performed in female C57BL/6 mice (Charles River) by oral inoculation of a 0.1 ml mixture containing equal numbers (1×10^8^ cfu) of a chloramphenicol resistant wild type strain (*ushA::Cm*) and mutant strains as described previously [5]. The marked wild type strain was previously shown to be phenotypically neutral [30]. Three days after infection, the spleen, liver and cecum was removed, homogenized in ice-cold PBS (Mixer Mill, Retsch) and serially diluted in PBS. The competitive index (CI) was determined by plating dilutions of the homogenized tissue lysates on agar plates containing streptomycin and incubating overnight at 37°C to recover both wild type and mutant bacteria. Colonies were then replica-stamped onto separate plates containing streptomycin and chloramphenicol to enumerate wild type and mutant bacteria. The CI was calculated as (cfu mutant/cfu wild type)_output_/(cfu mutant/cfu wild type)_input_. Mouse experiments were performed twice using groups of 5 mice for each experiment. Statistical analysis was performed using a Student *t* test.

## Conclusion

In summary, we have verified that SscA is the chaperone for the SseC translocon component in the T3SS encoded by SPI-2. This work completes the characterization of the known chaperone complement within SPI-2. In future work, it will be useful to investigate whether this particular chaperone-cargo pair has any additional regulatory function on gene expression within SPI-2.

## Abbreviations

SPI: *Salmonella* pathogenicity Island; T3SS: Type 3 secretion system; S. Typhimurium: *Salmonella enterica* serovar Typhimurium; TPR: Tetratricopeptide repeat; CI: Competitive index.

## Competing interests

The authors indicate that there are no competing interests.

## Author’s contributions

Conducted experiments and analyzed data: CAC, DTM, SEA. Wrote manuscript: CAC, DTM, BKC. Edited manuscript and provided essential discussion: CAC, DTM, SEA, AVCP, BKC. All authors read and approved the final manuscript.
